# Expression of complement components, receptors and regulators by human dendritic cells

**DOI:** 10.1016/j.molimm.2011.02.003

**Published:** 2011-05

**Authors:** Ke Li, Henrieta Fazekasova, Naiyin Wang, Pervinder Sagoo, Qi Peng, Wafa Khamri, Chantelle Gomes, Steven H. Sacks, Giovanna Lombardi, Wuding Zhou

**Affiliations:** King's College London, MRC Centre for Transplantation, NIHR Comprehensive Biomedical Research Centre, Guy's Hospital, London, UK

**Keywords:** Complement, Dendritic cell, Local production, Gene regulation

## Abstract

Integration of innate and adaptive arms of the immune response at a cellular and molecular level appears to be fundamental to the development of powerful effector functions in host defence and aberrant immune responses. Here we provide evidence that the functions of human complement activation and antigen presentation converge on dendritic cells (DCs). We show that several subsets of human DCs [i.e., monocyte derived (CD1a^+^CD14^−^), dermal (CD1a^+^DC-SIGN^+^), Langerhans (CD1a^+^Langerin^+^), myeloid (CD1c^+^CD19^−^), plamacytoid (CD45RA^+^CD123^+^)] express many of the components of the classical and alternative and terminal pathways of complement. Moreover human DCs have receptors known to detect the biologically active peptides C3a and C5a (C3aR, C5aR) and the covalently bound fragments C3b and metabolites iC3b and C3d which serve in immune adhesion (i.e., CR3, CR4, CRIg). We also show that the human DC surface is characterised by membrane bound regulators of complement activation, which are also known to participate in intracellular signalling (i.e., CD46, CD55, CD59). This work provides an extensive description of complement components relevant to the integrated actions of complement and DC, illuminated by animal studies. It acts as a resource that allows further understanding and exploitation of role of complement in human health and immune mediated diseases.

## 1 Introduction

Dendritic cells (DCs) and the complement system are two of the most important components of innate immunity, one being a cellular component and the other a humoral constituent. Recent research has shown that complement can influence the adaptive immune response by modulating DC function and thus regulating antigen specific T cell responses. In addition, local production and activation of complement has been suggested to be critical for DC functional development (Peng et al., 2006, 2008; Li et al., 2008; Baruah et al., 2009; Strainic et al., 2008).

DCs are potent antigen presenting cells (APCs) with the unique capacity to initiate the primary immune response. In addition to their role in local innate immune responses, DCs play a critical role in the adaptive immune response by priming the immune response or by inducing tolerance (Steinman, 2006). In humans, DCs with different phenotypes are distributed throughout the body and reside at the site of potential pathogen entry or tissue injury, where they differentiate into immature DCs. The diverse functions of DCs are dependent not only on their subsets but also on their state of activation which can be regulated by many mediators including exogenous and endogenous factors (Banchereau et al., 2000; Steinman, 2003).

The complement system consists of approximately 30 distinct proteins, including pathway components, receptors and regulators. The pathway components are a group of plasma proteins [e.g. C1–C9, factor B, factor D, mannose binding lectin (MBL) and MBL-associated serine proteases (MASPs)] which participate in complement activation through which complement effector molecules (e.g. C3b, C3a/C5a, C5b-9) are generated. The receptors are membrane proteins (e.g. CR1, CR2, CR3, CR4, C3aR and C5aR) which interact with their respective effector molecules. The regulators [e.g. factor I (fI), factor H (fH), properdin (fP), CD46, CD55, CD59] are either present in soluble form or expressed on cells, and are able to mediate several regulatory mechanisms to prevent tissue damage caused by complement activation. Complement receptors and membrane regulators have a wide cellular distribution (e.g. parenchymal cells and leukocytes including lymphocytes), whereas most of the soluble circulating complement components and regulators are thought to be mainly synthesised in the liver (Walport, 2001). However, extrahepatic production of complement has been shown in a variety of tissues/organs/cells which synthesise a number of complement proteins, either constitutively or in response to noxious stimuli (Li et al., 2007).

Although extrahepatic synthesis of complement has been widely studied in most cells and tissues, information on DCs has only recently emerged and most work has been confined to murine DCs (van Kooten et al., 2008). Previous studies have shown that murine bone-marrow derived DCs (BMDCs) are able to synthesise a range of complement pathway components, regulators and receptors and this synthesis has a substantial impact on DC activation, affecting DC functions in antigen presentation and allospecific T cell stimulation (Peng et al., 2006, 2008; Strainic et al., 2008). APCs from C1q-, C3-, fB- and fD-deficient mice exhibited a less activated phenotype and had a reduced capacity to stimulate antigen specific T cells (Peng et al., 2006; Li et al., 2008; Zhou et al., 2006; Heeger et al., 2005; Ghannam et al., 2008). In addition, DCs from C3aR or C5aR deficient mice have a similar activation and functional phenotype to the above complement component deficient mice, suggesting that C3aR- or C5aR-ligand interactions play a critical role in modulating DC activation and function (Li et al., 2008; Peng et al., 2008, 2009). These studies highlight the importance of local production of complement by APCs in modulating DC activation and function.

Although cell-autonomous expression of complement pathway components and receptors and regulators and their functional consequences have been documented in murine BMDCs, such information in human DCs is limited. Previous studies in man have found that several pathway components and regulators (e.g. C1q, C3, fI, fB, CR1) are expressed in monocyte-derived DCs (moDCs) (Castellano et al., 2004; Reis et al., 2006, 2007, 2008); and C1q and C3 can influence DC activation and maturation (Nauta et al., 2004; Csomor et al., 2007; Castellano et al., 2007), suggesting that human DCs, like murine DCs, have the capacity to synthesise complement and interact with it. However, several important questions remain, for example, whether moDCs can synthesise a diverse range of complement elements sufficient for local activation and detection of complement effectors; whether pathogen-associated or inflammatory stimuli regulate this synthesis; whether different subsets of DCs, such as dermal DCs (dDCs), Langerhans cells (LCs), plasmacytoid DCs (pDCs) and myeloid DCs (mDCs), have the same characteristics or exhibit different capacities to produce complement compared to moDCs.

In the present study, we addressed these questions by examining the expression of a wide range of complement pathway components, receptors and regulators using distinct subsets of human DCs (moDCs, dDCs, LCs, pDCs, mDCs) and the regulation of complement gene expression in moDCs by inflammatory or pathogen-related stimuli.

## 2 Materials and methods

### 2.1 Reagents for preparation of DCs

Microbeads for CD14, CD34, CD1a, and positive selection kits for BDCA-1 and BDCA-4 were purchased from Miltenyi (Surry, UK). GM-CSF, IL-4, IL-1 and TNF-α were purchased from Firstlink (Brierley Hill, UK). SCF and FLT3 ligand were purchased from Biosource Camarilio (CA, USA). TGF-β was purchased from R&D Systems Europe Ltd. (Abingdon, UK). LPS, Prostaglandin E_2_ (PGE_2_) was purchased from Sigma–Aldrich (Dorset, UK). RNA isolation kit was purchased from Applied Biosystems (Warrington, UK).

### 2.2 Samples

Collection of human samples (i.e., buffy coats, cord blood, peripheral blood) was approved by the local research ethics committee of Guy's Hospital, London, UK. Human buffy coats were obtained from NBS-South Thames, London, UK. Cord blood was obtained from King's College Hospital, which was approved by the local research ethics committee.

### 2.3 Preparation of human DCs

Monocyte-derived DCs were generated from peripheral blood monocytes by treatment with GM-CSF and IL-4. PBMC were isolated from buffy coat preparations from healthy donors by Ficoll-Hypaque gradient centrifugation, followed by anti-CD14 bead selection. CD14^+^ monocytes were cultured in RPMI-10% FCS, 2 mM l-glutamine, and 100 U/ml penicillin and streptomycin in the presence of GM-CSF (20 ng/ml) and IL-4 (20 ng/ml) at 37 °C in 5% CO_2_ atmosphere for 5 days. In some experiments CD14^+^ cells were cultured as described above but in the presence of 10% of human serum (HS) either normal (NS) or heat-inactivated (HIS) instead of FCS. In addition, in some cases moDCs were stimulated at day 5 with LPS (100 ng/ml) for 24 h, or day 4 with TIP (TNF-α, IL-1 both 20 ng/ml and PGE_2_ 2.5 × 10^−6^ M) for 48 h. Dermal DCs and LCs were generated from cord blood. CD34^+^ cells isolated by positive selection were cultured in RPMI supplemented by 10% FCS for 6 days, in the presence of SCF (25 ng/ml), FLT3 ligand (25 ng/ml), TNF-α (5 ng/ml) and GM-CSF (100 ng/ml), and followed by separation of CD14^+^ and CD14-cells. CD14^+^ cells (precursor of dDCs) were further cultured for 6 days, in the presence of GM-CSF (50 ng/ml), SCF (25 ng/ml), FLT3 ligand (25 ng/ml), IL-4 (1000 U/ml); while, CD14^−^ (precursor of LC) were further cultured in the presence of TNF-α (2.5 ng/ml) and TGF-β (5 ng/ml). CD1a^+^ dDCs and CD1a^+^ LC were isolated, respectively from the day 12 cultures by positive selection (Rozis et al., 2008). Plasmacytoid DCs were freshly isolated from peripheral blood by CD304 (BDCA-4) positive cell selection. Cells were then further sorted for CD123 (IL-3R) and CD303 (BDCA-2) expression. Sorted cells were then cultured overnight, in the presence of IL-3 (10 ng/ml). Myeloid DCs were freshly generated from peripheral blood by CD20^+^ depletion, followed by purification of CD1c^+^ cells using Miltenyi BDCA-1 positive selection kit.

### 2.4 Conventional RT-PCR

Total RNA was extracted from the cell pellets using Ambion RNA isolation kit and subsequently used for cDNA synthesis. cDNA synthesis was carried out with 5 μg of total RNA, 160 ng of oligo(dT)_12–18_, 500 μM of each dNTP, and 200 U Moloney murine leukemia virus reverse transcriptase in 20 μl of solution (50 mM Tris–HCl pH 8.3, 75 mM KCl, 10 mM DDT, 3 mM MgCl_2_, 1.5 U/ml RNasin) at 37 °C for 45 min. At the end of the reaction, cDNA was further diluted with sterile water (30 μl of water was added in 20 μl of reaction mixture) and stored at −20 °C until further use. PCR was carried out with 2 μl diluted cDNA (reflecting 0.2 μg of total RNA), 12.5 pmol of each 3′ and 5′ primer pair for each testing gene (the information for primer sequences are shown in supplementary Table) in 25 μl of reaction buffer (Promega, Southampton, UK). The PCR cycle consisted of 1 min at 94 °C, 1 min at 62 °C, and 1 min at 72 °C. Amplified PCR products were visualized after electrophoresis on 1.5% or 2% agarose gel containing ethidium bromide.

### 2.5 RT-quantitative PCR

RNA extraction and cDNA synthesis were performed as described in Section 2.4. RT-quantitative PCR (RT-qPCR) was performed with an MJ Research PTC-200 Peltier Thermal Cycler and DyNAmo™ HS SYBR^®^ Green qPCR kit (MJ BioWorks, Finland). PCR was set up in low-profile microplates containing 10 μl of master mix, 1 μl diluted cDNA (reflecting 0.1 μg of total RNA), 5 pmol of each 3′ and 5′ primer pair, either for each testing gene or 18 s gene, in 20 μl reaction volume. Amplification was performed according to manufacturer's cycling protocol and done in triplicate. Gene expression was expressed as 2^−ΔΔ(Ct)^ (Pfaffl et al., 2002), where Ct is cycle threshold, ΔΔ(Ct) = sample 1 Δ(Ct) − sample 2 Δ(Ct); Δ(Ct) = 18 s (Ct) − testing gene (Ct).

### 2.6 Flow cytometric analysis

The following monoclonal anti-human antibodies were used for the detection of complement expression by flow cytometry: FITC anti-CR3, -CR4 (Serotec, Oxfordshire, UK); PE anti-C3aR, -C5aR, -CD55 and -CD59 (Biolegend, San Diego USA); mouse anti-CD46 (GB24) (a gift from Dr John Atkinson, Washington University School of Medicine, St. Louis, USA) which was combined with FITC anti-mouse IgG.

### 2.7 Western blot

The cell lysate prepared from moDCs was separated onto SDS–PAGE followed by electro blotting. Membranes were incubated with the antibodies against each detecting molecules and then peroxidase conjugated anti-rabbit or anti-goat IgG. Immunoblots were developed using ECL Western Blotting Detection Reagents. The following polyclonal anti-human antibodies were used for the detection of complement expression: rabbit anti-C2; goat anti-fI, -fH, -fB, and -fD (Quidel, San Diego, USA); goat anti-C1r and -C1s (Source Bioscience LifeSciences, Nottingham, UK); rabbit anti-propordin (a gift from Professor Wilhelm Schwaeble Leicester University, UK); peroxidise conjugated anti-rabbit or anti-goat IgG (Dako, Cambridgeshire, UK).

### 2.8 ELISA

The supernatants collected from 24 h DC cultures, in the presence or absence of LPS, were used for analysing C1q, C3 and C4 by sandwich ELISA. In brief, 96-well plates (Nunc) were coated overnight at 4 °C with sheep anti-human C3c (1/200) (The Binding Site), mouse anti-human C1q (1/200) (Quidel, San Diego, USA), mouse anti-human C4c (1/200) (Quidel) respectively. After blocking with PBS containing 1% BSA, the plate was incubated with appropriately diluted test samples, followed by rabbit anti-human C3c, C1q or C4c (all from Dako, 1/3000), then peroxidase conjugated goat anti-rabbit IgG (1/5000) (Dako). Each antibody incubation was performed in 100 μl PBS containing 1% BSA, 0.1% Tween at 37°C for 1 h and followed by washing in PBS containing 0.1% Tween. The enzyme activity was read after incubation with *O*-phenylenediamine by measuring absorbance at 490 nm. Purified human C3, C1q, C4 (Quidel) were used to generate a standard curve.

## 3 Results

### 3.1 Expression of complement components, receptors and regulators by moDCs

**Fig. 1:**
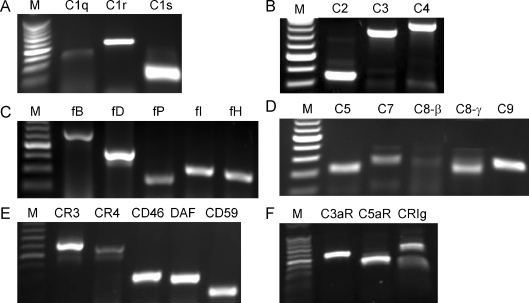
Gene expression of complement components, receptors and regulators by moDCs. Conventional RT-PCR was performed in moDCs without further stimulation after 6 days culture. The agarose gels show the PCR products for early components [C1q, C1r, C1s C2, C3, C4, fB, fD, fP (properdin)], soluble regulators (fI, fH) (A–C), late components (C5, C7, C8β and γ subunits, and C9) and membrane receptors/regulators (CR3, CR4, CD46, CD55, CD59, C3aR, C5aR and CRIg (D–F). The 100 bp DNA markers (M) are shown alongside the gels. A representative of 3 independent experiments with separate cell preparations is shown.

**Fig. 2:**
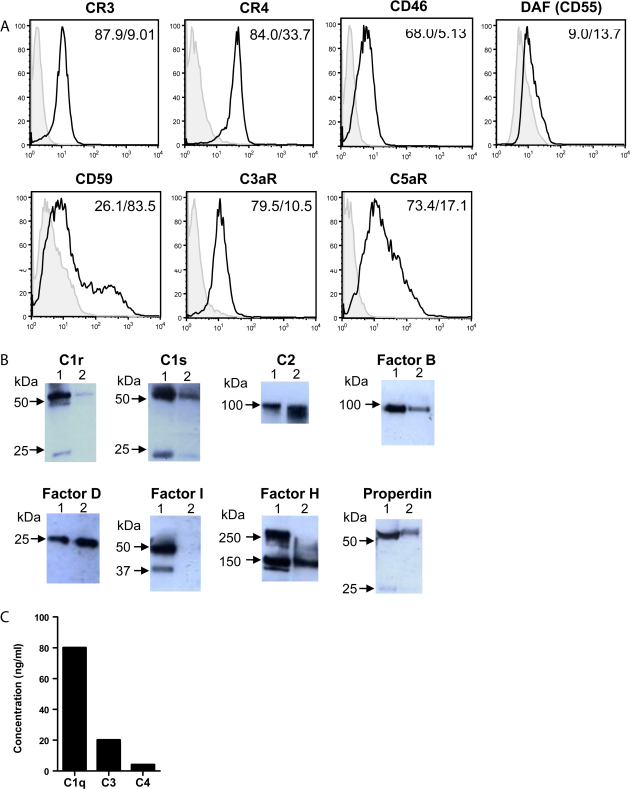
Protein synthesis of complement components, receptors and regulators by moDCs. 6 day cultured moDCs were used for protein analysis. (A) Flow cytometry. The numbers describe percentage of positive cells and mean fluorescence intensity for given marker. (B) Western blot. The supernatant collected from 24 h DC cultures were used for the assay. Lane 1 in each blot is positive control, where human serum was used for C1r and C1s, fP, fI, fH, C2, fB, fat tissue for fD; lane 2 in each blot is DC supernatant. (C) ELISA. The supernatants collected from 24 h DC cultures were used for analysing C1q, C3 and C4 by sandwich ELISA, the ranges of proteins detected in our 3 experiments are 30–80 ng/ml for C1q, 2–20 ng/ml for C3 and 1–4 ng/ml for C4. A representative of 3 independent experiments with separate cell preparations is shown.

We initially focused our study on monocyte derived DCs (moDCs), as they are the most common type of DC reported in human studies. In our study, moDCs were characterized by expression of CD1a and CD11c and low levels or absence of CD14. In addition, moDCs expressed MHC-class I and -class II, CD40 and CD86 (sFig. 1). To determine the ability of moDCs to synthesize complement components, receptors and regulators, we combined RT-PCR and protein assays. The results showed that mRNA transcripts for most complement components (i.e., C1q, C1s, C1r, C2, C3, C4, C5, C8, C9, fB, fD), regulators [i.e., fI, fH and fP (properdin), CD46, CD55, CD59] and receptors (i.e., CR3, CR4, C3aR, C5aR, CRIg) were detected in moDCs after 6 days of culture, in the absence of any stimulation (Fig. 1). Proteins for these mRNA transcripts except for fI were also detected in these cells by flow cytometry, western blot analysis or ELISA (Fig. 2). Thus, moDCs under steady state conditions are able to produce a wide range of complement components, receptors and regulators. This suggests that moDCs are not only a significant local source of complement, but also capable of detecting soluble (C3a and C5a) or membrane bound (C3b and its metabolite iC3b) complement effector molecules.

### 3.2 Regulation of complement expression in moDC by LPS, inflammatory stimuli and serum complement

**Fig. 3:**
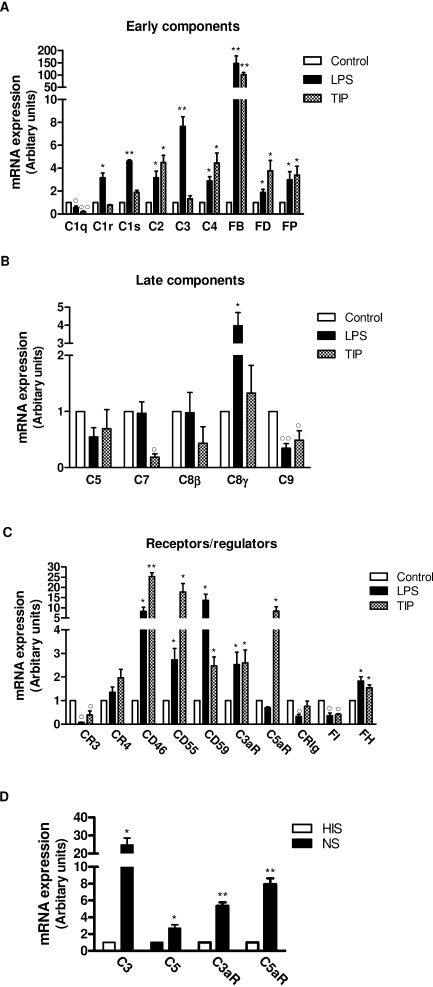
Regulation of complement in moDCs by LPS or inflammatory stimuli and serum complement. (A–C). Real-time PCR was performed in moDCs with 24 h LPS or 48 h TIP stimulation to quantify the level of gene expression for complement components, receptors and regulators. As described in Section 2, relative RNA levels were determined using the Δ(Ct) value for untreated DCs samples as a baseline for comparison. Each value represents the mean (±SEM) of three independent experiments. Data were analyzed by Student *t* test. *P* values are for comparisons between with or without treatment (°, *P < 0.05;°°, **P < 0.01). (D) Real-time PCR was performed in DCs treated with either normal (NS) or heat-inactivated serum (HIS) for 6 days to quantify the level of complement gene expression. Relative RNA levels were determined using the Δ(Ct) value for HIS-treated DCs samples as a baseline for comparison. Data were analyzed by Student *t* test. *P* values are for comparisons between NS or HIS treatment (*P < 0.05; **P < 0.01).

Previous studies have shown that diverse cellular functions of moDCs can be regulated by various exogenous and endogenous factors such as LPS, cytokines and steroids (Fazekasova et al., 2009; Buckland and Lombardi, 2009). Therefore, we asked whether the expression of complement in moDCs could be regulated by mediators that can be generated during infection and tissue inflammation [i.e., LPS and TNF-α/IL-1/PGE_2_ (TIP)]. We performed real time RT-PCR on LPS or TIP treated moDCs to quantify the level of gene expression for complement components, receptors and regulators. The results showed that LPS or TIP stimulation up-regulated the expression of most early components (C2, C3, C4, C5, fB, fD, fP), particularly fB, which increased >100-fold by both LPS and TIP stimulation, and C3, which increased by 7-fold by LPS stimulation. However, C1q was down-regulated by both LPS and TIP stimulation. LPS and TIP stimulation had relatively small effects on the expression of late components, in that C8γ was up-regulated by LPS, while C7 and C9 were down-regulated by LPS or/and TIP. LPS or TIP treatment led to increased expression of several receptors (C3aR, C5aR) and regulators (CD46, CD55, CD59, fH); however, LPS and/or TIP down-regulated CR3, fI and CRIg expression (Fig. 3A–C). These results indicate that expression of complement components/receptors/regulators is differentially regulated by LPS and other inflammatory stimuli.

In addition to pathogen-related and inflammatory mediators, we examined the effect of complement activation on the expression of several key complement components and receptors, as DCs differentiated from monocytes *in vivo* may be exposed to complement produced either locally or in the circulation. MoDCs were cultured in medium supplemented with 5% of either normal serum (with intact complement) or heat-inactivated (no complement activation) for 6 days, after which expression of C3, fB, C5, C3aR and C5aR was analyzed by real-time RT-PCR. We found that expression of C3, C5 and receptors for C3a and C5a was significantly higher in DCs differentiated in normal serum than in heat-inactivated serum (Fig. 3D). These results suggest that complement activation itself can lead to auto-amplification of the key complement components and receptors expressed in the inflammatory environment.

### 3.3 Complement gene expression in subsets of DCs

In addition to moDCs, there are several subsets of human DCs, including dDCs and LCs, pDCs and mDCs (Shortman and Liu, 2002). As subsets of DCs are specialized cells that arise from separate developmental pathways, preferentially located at different compartments (e.g. peripheral tissue, secondary lymphoid organ, bone marrow and circulation) and performing different functions, it is important to understand whether they have the same ability as moDCs to synthesize complement, since this may have implications for modulating their functions by complement activation. Therefore, we investigated the expression of complement components, receptors and regulators in dDCs, LCs, pDCs and mDCs. Dermal DCs and LCs were generated *in vitro* from cord blood CD34^+^ cells by treatment with specific cytokines and growth factors. The dDCs were characterized by expression of DC-SIGN, CD1a, CD11b, while LC assignment was based on the expression of langerin and CD1a (sFig. 2). Plasmacytoid DCs (CD304^+^ CD123^+^ CD303^+^) and mDCs (CD20^−^ CD1c^+^) were freshly isolated from peripheral blood (sFig. 2). We performed real time RT-PCR in the prepared subsets of DCs and found that, like the moDCs, the four subsets of DCs expressed a wide range of complement components, receptors and regulators. In general, the expression patterns were similar between moDCs and the other subsets of DCs studied, although there were differences in the levels of expression for some genes. Noticeable differences included that of C1q, which was not detected in the other four DC subsets, although higher levels of CD46, CD55 and C5aR were detected in those subsets of DC (Tables 1 and 2). These results suggest that although differences were observed between human DC subsets, the ability to synthesize multiple complement components/receptors/regulators is a feature of human DCs, extending previous observation (Reis et al., 2006). Furthermore, human DCs, like murine DCs, not only contribute to the local complement pool, but also potentially detect complement effector molecules through their complement receptors/regulators, which in turn modulate DC activation and function.

## 4 Discussion

Our previous work found that murine BMDCs are an important local source of complement and can detect activated complement products (e.g. C3a, C5a) through specific receptors (e.g. C3aR, C5aR) expressed on DCs, which leads to cell activation and functional modulation. The present study not only extends our previous findings to human moDCs, but also identifies several subsets of human DCs with the ability to synthesize the complement pathway components, receptors and regulators, suggesting that complement mediated modulation of DC function represents a generalised mechanism for regulation of T cell immunity.

DCs reside in peripheral tissues, particularly in the interstitial space at the site of encounter with pathogens and tissue stress factors, where it seems there may be insufficient access for complement components derived from the circulating pool, particularly the large molecules such as C1q, C3, C5 (>180 kDa). In this case, local synthesis of complement components by APCs may be an important factor which modulates APC function. Our findings that human moDCs and several other DC subsets can produce complement pathway components and express receptors that detect complement effector molecules, are largely supportive of this notion. In particular, when taken into consideration with the impairment of APC function reported in mice with C1q, fB, fD, C3, C3aR or C5aR deficiency and in an individual with complete C3 deficiency (Peng et al., 2006, 2008, 2009; Li et al., 2008; Ghannam et al., 2008), our results suggest that early effector molecules (e.g. C1q, C3a and C5a) generated by this means could play an important roles in human DC functional modulation. On the other hand, our results show that mRNA transcripts for several complement components, regulators and receptors including MASP (1 and 2), C6, C8 (α and β) were not detected in our system, although were clearly detected in positive control samples (sFig. 3). The lack of expression of MASPs and C6, C8 (α and β) may suggest that DC expression does not favour the activation of complement through the mannose binding lectin pathway and the formation of C5b-9 which could otherwise mediate the destruction of autologous DCs. It should be noted, however, that since DCs coexist with other cell types capable of secreting complement into the local environment, including endothelial and epithelial cells, it is likely that neighbouring cells also contribute to the local pool of complement as well as complement mediated modulation of APC function.

We have carried out extensive studies on complement gene expression and its regulation by LPS and TIP in moDCs. LPS was chosen to mimic bacterial infection, while TIP was selected to reflect non-pathogen related inflammatory conditions. Our data showed that moDCs with or without LPS or TIP stimulation express a wide range of complement pathway components, regulators and receptors, indicating that production of complement is an important feature of DCs under both physiological and pathological conditions. In addition, following LPS or TIP stimulation, the expression of C3, fD and fP and particularly fB, was significantly increased; however expression of fI (a complement inactivator) was decreased. Furthermore, several complement receptors (C3aR, C5aR, CR3, CR4) were clearly detected in moDCs, suggesting that moDCs can interact with various complement effector molecules (C3a, C5a, C3b, iC3b) through their receptors. Constitutive expression of complement membrane regulators (CD46, CD55 and CD59) and their up-regulation by LPS and TIP suggest that moDCs can be protected from complement mediated lysis, especially under pathological conditions that involve excessive complement activation. Expression of CD46 and CD55 also suggests DCs may interact not only with complement effectors, but also some pathogens, as both membrane regulators have been shown to bind to certain virus and bacteria (Liszewski et al., 2005; Hourcade et al., 2000).

In addition to RT-PCR, we have performed protein analysis in moDCs for those of their genes that were positively detected by RT-PCR. Our data shows that protein expression could be observed for all the complement components studied in moDCs, except for fI. The discrepancy between the mRNA and the protein detection could be due to the very low level expression of this factor or alternative that factor I was degraded. The protein analysis was not performed in other subsets of human DCs because the restriction of the cell number available. However, based on the published observations that for many complement related genes identified by RT-PCR, their corresponding proteins are detectable (Zhou et al., 1993; Sacks et al., 1993; Gasque et al., 1995; Gerritsma et al., 1997; Harris et al., 1999), together with the results using moDCs in the present study, we anticipate that the gene expression of complement detected by RT-PCR in the other subsets of human DCs will be inevitably accompanied by protein expression.

In conclusion, we have produced a catalogue of the capabilities of human DCs to function cooperatively with complement. Although we have not tested the functional capacity of DCs in the present study, the close similarities with mouse DCs, which exhibit a similar array of complement components and sensing molecules as the human cells described here, strongly suggests that human DCs will have the same functional dependence on complement. Thus, we anticipate that the ability of human DCs to present antigen to naïve T cells and influence T cell differentiation will depend on complement activation, and in particular the generation and sensing of C3a and C5a in the local environment. These studies form the subject of ongoing work.

## Disclosures

None.

## Figures and Tables

**Table 1:** Gene expression of complement components in human moDCs and subsets of DCs.

	Mono DC	Dermal DC	Langerhans cell	Myeloid DC	Plasmacytoid DC
C1q	+	−	−	−	−
C1r	+	++	+	±	±
C1s	+	++	+	+	−
C2	++	+	+	+	−
C3	+	++	±	+	+
C4	+	++	+	++	+
C5	++	+++	++	+++	++
C7	±	−	+	+	−
C8-β	±	+	+	++	+
C8-γ	+	+	+	++	±
C9	+	−	±	±	−
fB	+	++	+	++	++
fD	++	++	+++	+++	+
fP	**++**	++	++	++	++

Relative expression of RT-PCR product obtained with RNA from subsets of DCs based on 3 preparations. ± = occasionally detected, + = weakly detected, ++ = detected, +++ or ++++ = strongly detected. C1q sequences are derived from the b chain of C1q.

**Table 2:** Gene expression of complement receptors/regulators in human moDCs and subsets of DCs.

	Mono DC	Dermal DC	Langerhans cell	Myeloid DC	Plasmacytoid DC
CR1	±	++	+	++	±
CR2	−	+	+	++	+
CR3	++	++	+++	++	−
CR4	+	+	+	+	++
CD46	++	+++	+++	+++	+++
CD55	++	+++	++++	+++	+++
CD59	++	++	+++	+	+
C3aR	++	++	++	++	++
C5aR	+	++	++	+++	+++
CRIg	+	+	+	++	+
fI	+	++	+	++	+
fH	++	++	+++	+	+

Relative expression of RT-PCR product obtained with RNA from subsets of DCs based on 3 preparations. ± = occasionally detected, + = weakly detected, ++ = detected, +++ or ++++ = strongly detected. C1q sequences are derived from the b chain of C1q.
