# Supplementary material for: Expression of complement components, receptors and regulators by human dendritic cells
Source: Mol Immunol. 2011 May;48(9-10):1121–7. doi: 10.1016/j.molimm.2011.02.003 (PMC3084445; doi:10.1016/j.molimm.2011.02.003)
Supplement: Supplementary file 1 [file mmc1.ppt]

## Slide 1
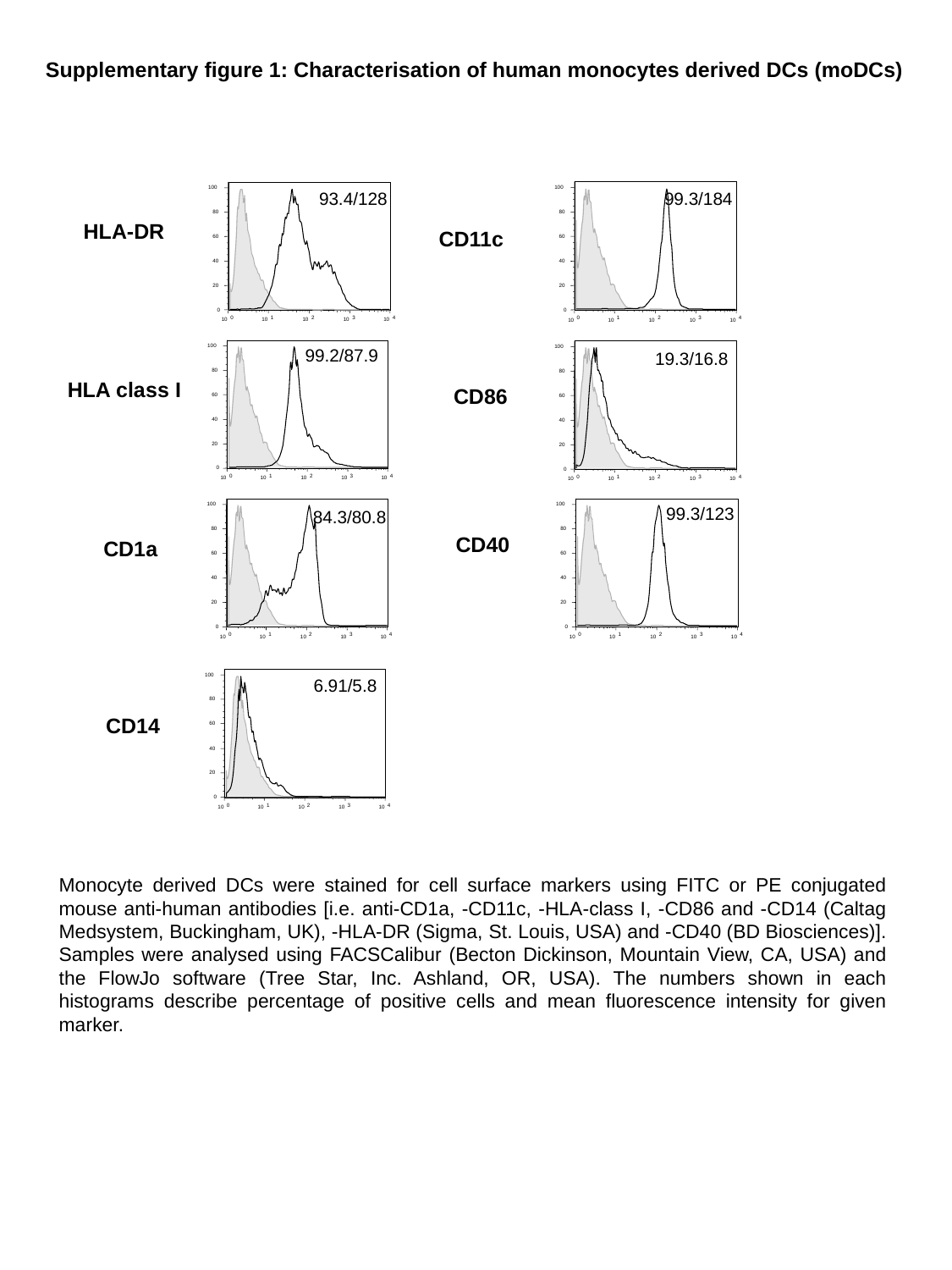

Supplementary figure 1: Characterisation of human monocytes derived DCs (moDCs)
99.3/184
100
80
60
40
20
0
0
1
2
3
4
10
10
10
10
10
93.4/128
100
80
60
40
20
0
0
1
2
3
4
10
10
10
10
10
HLA-DR
CD11c
99.2/87.9
100
80
60
40
20
0
0
1
2
3
4
10
10
10
10
10
100
80
60
40
20
0
0
1
2
3
4
10
10
10
10
10
19.3/16.8
HLA class I
CD86
99.3/123
100
80
60
40
20
0
0
1
2
3
4
10
10
10
10
10
100
80
60
40
20
0
0
1
2
3
4
10
10
10
10
10
84.3/80.8
CD40
CD1a
6.91/5.8
100
80
60
40
20
0
0
1
2
3
4
10
10
10
10
10
CD14
Monocyte derived DCs were stained for cell surface markers using FITC or PE conjugated mouse anti-human antibodies [i.e. anti-CD1a, -CD11c, -HLA-class I, -CD86 and -CD14 (Caltag Medsystem, Buckingham, UK), -HLA-DR (Sigma, St. Louis, USA) and -CD40 (BD Biosciences)]. Samples were analysed using FACSCalibur (Becton Dickinson, Mountain View, CA, USA) and the FlowJo software (Tree Star, Inc. Ashland, OR, USA). The numbers shown in each histograms describe percentage of positive cells and mean fluorescence intensity for given marker.

## Slide 2
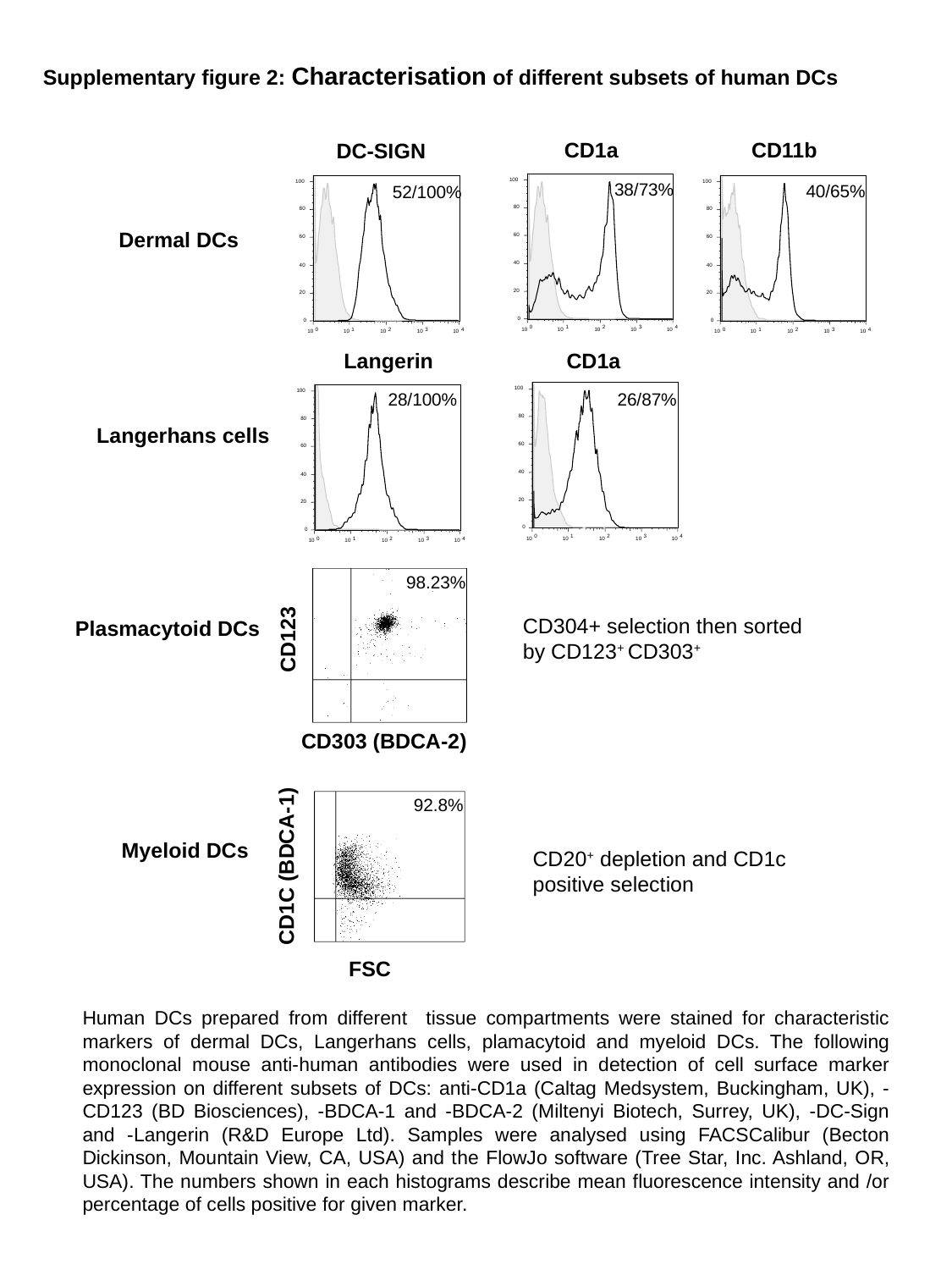

Supplementary figure 2: Characterisation of different subsets of human DCs
CD1a
CD11b
DC-SIGN
38/73%
100
80
60
40
20
0
0
1
2
3
4
10
10
10
10
10
40/65%
100
80
60
40
20
0
0
1
2
3
4
10
10
10
10
10
52/100%
100
80
60
40
20
0
0
1
2
3
4
10
10
10
10
10
Dermal DCs
Langerin
CD1a
100
80
60
40
20
0
0
1
2
3
4
10
10
10
10
10
26/87%
28/100%
100
80
60
40
20
0
0
1
2
3
4
10
10
10
10
10
Langerhans cells
CD123
CD303 (BDCA-2)
98.23%
CD304+ selection then sorted by CD123+ CD303+
Plasmacytoid DCs
CD1C (BDCA-1)
FSC
92.8%
Myeloid DCs
CD20+ depletion and CD1c positive selection
Human DCs prepared from different tissue compartments were stained for characteristic markers of dermal DCs, Langerhans cells, plamacytoid and myeloid DCs. The following monoclonal mouse anti-human antibodies were used in detection of cell surface marker expression on different subsets of DCs: anti-CD1a (Caltag Medsystem, Buckingham, UK), -CD123 (BD Biosciences), -BDCA-1 and -BDCA-2 (Miltenyi Biotech, Surrey, UK), -DC-Sign and -Langerin (R&D Europe Ltd). Samples were analysed using FACSCalibur (Becton Dickinson, Mountain View, CA, USA) and the FlowJo software (Tree Star, Inc. Ashland, OR, USA). The numbers shown in each histograms describe mean fluorescence intensity and /or percentage of cells positive for given marker.

## Slide 3
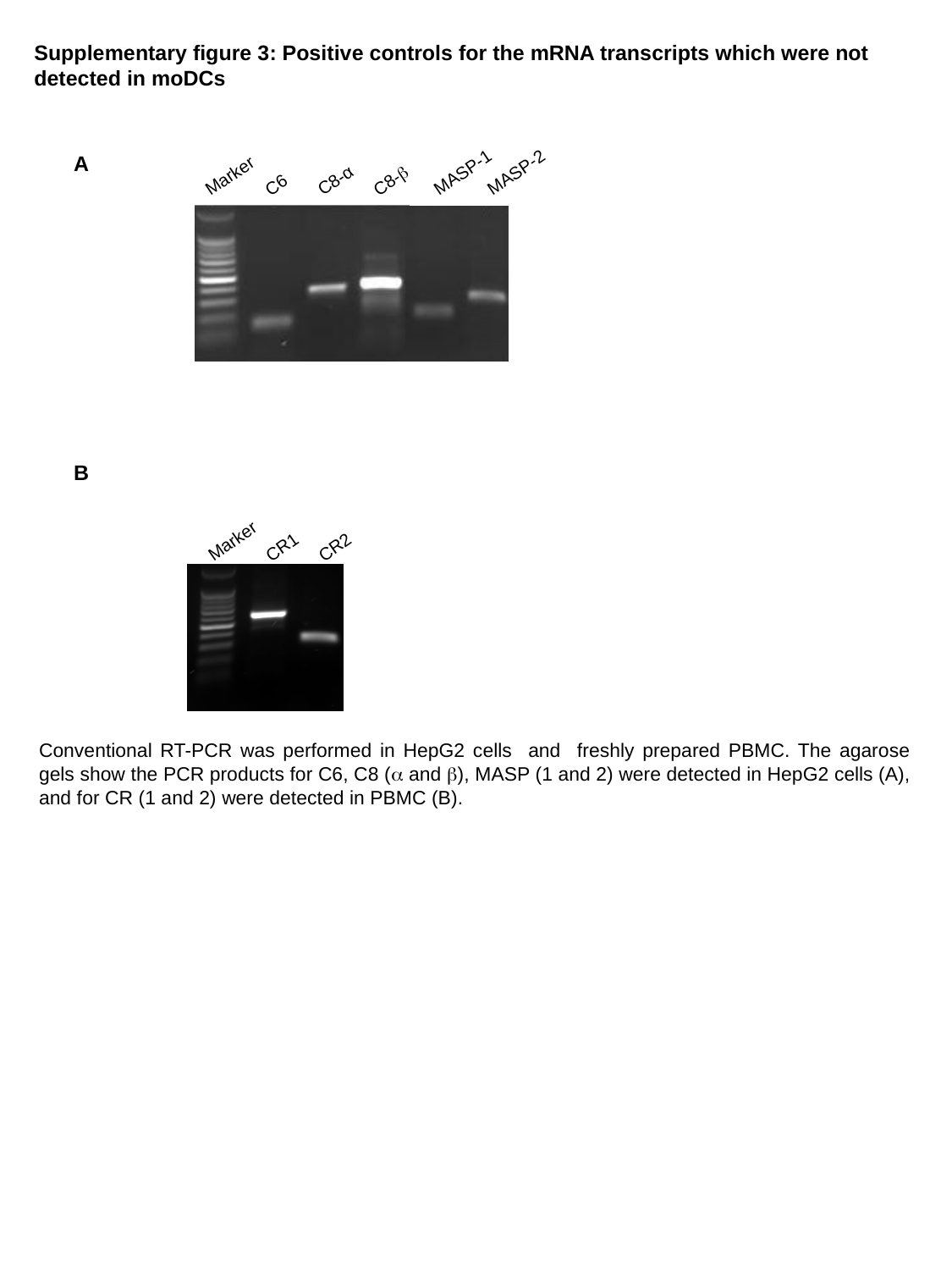

Supplementary figure 3: Positive controls for the mRNA transcripts which were not detected in moDCs
A
MASP-1
MASP-2
Marker
C8-α
C8-β
C6
B
Marker
CR1
CR2
Conventional RT-PCR was performed in HepG2 cells and freshly prepared PBMC. The agarose gels show the PCR products for C6, C8 (α and β), MASP (1 and 2) were detected in HepG2 cells (A), and for CR (1 and 2) were detected in PBMC (B).
