# Supplementary material for: Expression of complement components, receptors and regulators by human dendritic cells
Source: Mol Immunol. 2011 May;48(9-10):1121–7. doi: 10.1016/j.molimm.2011.02.003 (PMC3084445; doi:10.1016/j.molimm.2011.02.003)
Supplement: Supplementary file 2 [file mmc2.doc]

**Supplemental Table**

# PCR primer sequences and product sizes

| Primer* | **Oligonucleotide Sequence**  **(5’ → 3’)** | **Product Size (bp)** | **Gene bank code** |
| --- | --- | --- | --- |
| C1qb-1 | CAGGGATAAAAGGAGAGAAAGG | 357 | X03084 |
| C1qb-2 | TGGCGTGGTAGGTGAAGTAGTA |  |  |
| C1r-1  C1r-2 | GATCTATGCCAACGGGAAGA  CATTCTTCCAAATGCCCTGT | 545 | NM_001733.4 |
| C1s-1  C1s-2 | AAGAGCGTTTTACGGGGTTT  AATCTCCCCAATCAGTGCAG | 212 | NM_001734 |
| C2-1 | CCTTGAATGGGAGCAAACTGAAC | 339 |  |
| C2-2 | GATTGATGTGAAAGTCTCGTGGC |  | X04481 |
| C3-1 | GCTGCTCCTGCTACTAACCCA | 784 | K02765 |
| C3-2 | AAAGGCAGTTCCCTCCACTTT |  |  |
| C4-1 | TGCGGATCCAGCAGTTTCGG | 889 | K02403 |
| C4-2 | TGGCGGTTGTTCAGCTGCAG |  |  |
| FB-1  FB-2 | GTG TGA CCA CCA CTC CAT GG  CCA TCC TCA GCA TCG ACT CC | 685 | L15702 |
| MASP1-1  MASP1-2 | Gctggaggctctcatacagg  acgtcccatccttcagacac | 198 | NM_001879 |
| MASP2-1  MASP2-2 | Gctccgactactccaacgag  ggctgatgctgtaagtgcaa | 297 | NM­_006610 |
| FD-1  FD-2 | CGACCACGACCTCCTGCTGCTACA  GCTCGGGACTTTGTTGCTTGGGTG | 479 | M84526 |
| FH-1  FH-2 | ACATTACTTCATTCCCGTTGTC  ATACTCCAGTTTCCCATCCCAA | 320 | Y00716 |
| FI-1  FI-2 | TGGGAGGAAAGCGAGCACAACT  CACAGGCAGGGATGGAACGAGG | 339 | Y00318 |
| Properdin-1  Properdin-2 | GATGGGCGGCTGGTCTGGCTG  TGCGGCTTCGTGTCTCCTTAG | 263 | X57748 |
| C5-1 | AGTGTGTGGAAGGGTGGAAG | 222 | NM_001735 |
| C5-2 | GTTCTCTCGGGCTTCAACAG |  |  |
| C6-1 | tggggtcttgaaaggacaag | 153 | NM_000065.2 |
| C6-2 | gagttggtttccacccttga |  |  |
| C7-1 | aaaatgccctacgaatgtgg | 261 | NM_000587 |
| C7-2 | aaacccttcttcctcgcact |  |  |
| C8α-1  C8α -2 | Cggtatacaatggggaatgg  gcagtctgcacctttgtgaa | 361 | NM_000562 |
| C8β-1  C8β-2 | Caaagaggccatggagagag  tgcttcatgttctgcctcac | 390 | NM_000066 |
| C8γ-1  C8γ-2 | Ccagagtttcgctgtcctgt  cctcacctcctcacttcgtc | 216 | NM_000606 |
| C9-1 | caactgggcctcttccataa | 251 | NM_001737 |
| C9-2 | cacaggcaattccctcaaat |  |  |
| CR1-1 | AGTCATTGTGTCTTGGTTGG | 609 | XM_015278 |
| CR1-2 | AGACGAGACAAGTAGTAGATGG |  |  |
| CR2-1  CR2-2 | TTCTCCTGGAATGTCAATCC  AAGGACAGGAGCAAGTGAACGG | 321 | XM_002008.3 |
| CR3-1  CR3-2 | AAGTGTGTATGTGCGTGTGTGC  CCATAGACAGGCTTATCCATCC | 617 | NM_000632 |
| CR4-1  CR4-2 | GCTGCAAGCATCATTCGTTA  CCAATCAGGACCAGGTCAGT | 575 | NM_050142.1 |
| C3aR-1  C3aR-2 | ACTCGTGGAGACATCCAGGT  GAAGATTTCCCGGTACACGA | 555 | NM_004054 |
| C5aR-1  C5aR-2 | GAGCCCAGGAGACCAGAACATG  TACATGTTGAGCAGGATGAGGGA | 441 | NM_001736 |
| CRIg-1  CRIg-2 | ccatatccagcaggca  catccatgtcagtggt | 581(L)  348(S) | NM-007268 |
| DAF-1 | TACTACCCGTCTTCTATCTGGG | 295 | NM-000574 |
| DAF-2 | TTTTCAAGAGGTGTAGGTGTGC |  |  |
| MCP-1 | GCTGCTCCAGAGTGTAAAGTGG | 308 | XM_015281 |
| MCP-2 | AACAATCACAGCAATGACCC |  |  |
| CD59-1 | actgcaaaacagccgtcaat | 210 | NM_000611 |
| CD59-2 | aggatgtcccaccattttca |  |  |
| 18S-1 | GACTCAACACGGGAAACCTC | 153 | NM_011296.1 |
| 18S-2 | ATGCCAGAGTCTCGTTCGTT |  |  |

* Primer-1 is identical to the coding strand; primer-2 is complementary to the coding strand. All primers were designed such that there are intronic sequences between the primer 1 and primer 2.
